# Critical Lengths of Kitaev Chains for Majorana Zero Modes with a Microsecond Coherence Time and a Quantized Conductance Signature

**DOI:** 10.3390/ma17235898

**Published:** 2024-12-02

**Authors:** Mirko Poljak

**Affiliations:** Computational Nanoelectronics Group, University of Zagreb Faculty of Electrical Engineering and Computing, HR 10000 Zagreb, Croatia; mirko.poljak@fer.unizg.hr

**Keywords:** Kitaev chain, topological superconductor, nanowire, Majorana zero mode, coherence time, quantum transport, transmission, conductance, zero-bias conductance peak, critical length, non-equilibrium Green’s function (NEGF)

## Abstract

The problems of disorder and insufficient system length are generally regarded as central problems in the realization of Majorana zero modes (MZM), which are a promising platform for realizing fault-tolerant topological quantum computing (TQC). In this work, we analyze eigenenergy spectra and transport properties of finite Kitaev chains using quantum transport simulations in a wide design space of hopping amplitude (*t*), superconductor pairing (Δ), and electrochemical potential. Our goal is to determine critical or minimum acceptable chain lengths to obtain oscillation-free MZMs with suitable microsecond coherence times, and observable zero-bias conductance peaks (ZBCP) quantized almost at ~2*e*^2^/*h*. Due to qualitative equivalence of the Kitaev and Oreg–Lutchyn models, we approximately determine the foreseeable critical length of topological superconducting nanowires (TS NWs) as well. We find that the ZBCP length requirement is looser in comparison to the limit imposed by the coherence time. For a large *t*/Δ mismatch of ~40 corresponding to the experimental TS NWs, the first condition sets the minimum length to 344 sites (≈5.5 μm), while the second condition requires 605 sites (≈9.7 μm). The calculated lengths are far from the reported experimental hybrid device dimensions, explaining difficulties in observing MZMs in TS NWs fabricated so far. Nonetheless, a decreasing *t*/Δ mismatch allows for shorter systems, which argues in favor of the proximitized quantum dot path for MZMs in a solid-state system.

## 1. Introduction

Topological quantum computing (TQC) uses anyons, which are quasiparticles with generalized statistics, for quantum information processing [1,2]. The exchange or braiding of anyons is a topological quantum evolution as it depends on local geometrical details. Moreover, a special type of anyons—non-Abelian or non-commutative anyons—enables fault-tolerant quantum computation, since the non-local topological property makes them robust to (weak) local perturbations. Anyons can form a degenerate subspace on which TQC is performed by braiding, i.e., by physically moving anyons around each other. In the case of non-Abelian anyons, the final result of a TQC operation depends on the order in which anyon exchanges are performed. Non-Abelian anyons can be realized in different ways. The simplest approach is to create a quasiparticle that supports a Majorana zero mode (MZM) in a one-dimensional (1D) topological superconductor (TS) [3,4].

A toy model of a 1D TS was proposed by Kitaev [1] for spinless fermions with *p*-wave superconducting pairing, who demonstrated the ability of the so-called 1D Kitaev chain to support MZMs at its ends. This configuration in turn enables the characterization of MZMs by tunneling conductance measurement. Further theoretical considerations proposed a realistic 1D TS system for experimental demonstration based on a heterostructure consisting of a topological insulator and a superconductor [2]. Mourik et al. [3] provided indications for MZMs by measuring the zero-bias conductance peaks in InSb nanowires (NW) with proximitized superconductivity in a strong magnetic field. However, unambiguous evidence of MZMs, localization and braiding features have not yet been demonstrated in TS NWs, mainly due to finite temperature effects, the presence of defects and impurities, various sources of disorder, etc. [4,5]. Another proposal for a 1D TS system is a chain of coupled quantum dots (QD) [6], for which Dvir et al. [7] and Zatelli et al. [8] have recently demonstrated the existence of the so-called “poor man’s Majorana states”. Further upscaling to a longer QD Kitaev chain is required for topological protection and attenuation of the noise affecting the tunnel-coupling rates [7]. Therefore, the issue of system length is central to both the TS NW and QD chain approaches to MZMs and the associated TQC.

In this paper, we provide a device-engineering view on the design space constraints for finite Kitaev chains by analyzing eigenenergy spectra and transport properties using the non-equilibrium Green’s function (NEGF) formalism. We analyze the fundamental properties of MZMs and investigate how they change for different chain lengths when the design parameters are altered, including the variation of hopping amplitude, superconductor pairing, and electrochemical potential. Our results are generally applicable to TS NWs as well, due to qualitatively equivalent physics described by the Kitaev [1] and Oreg–Lutchyn [9,10] models. While TS NWs of different lengths have been studied in the past in terms of their suitability for MZMs and TQC [11,12,13,14,15], our effort focuses on finding the critical or minimum acceptable chain length that leads to oscillation-free MZMs with coherence times of at least ~1 μs. Furthermore, we investigate the transport properties, including the Andreev processes, and analyze the contributions and total transmission as well as the zero-bias conductance at 0 K. From the transport analysis, we determine the minimum acceptable Kitaev chain lengths for observing the nearly perfectly quantized local conductance of ~2*e*^2^/*h* under zero-bias conditions. We find that the critical length requirement is a likely “nail in the coffin” for observing MZMs in TS NWs and a likely source of optimism for the QD idea.

## 2. Theory and Methodology

**Figure 1:**
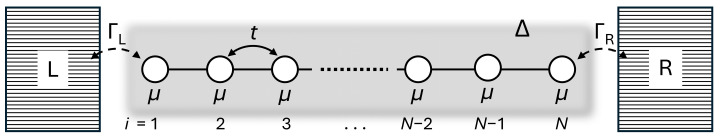
Illustration of an *N*-site Kitaev chain with definitions of the electrochemical potential (*μ*), nearest-neighbor hopping parameter (*t*), superconductor pairing (Δ), and tunneling contacts (Γ*_L_*, Γ*_R_*).

Majorana modes can be found at topological domain walls, i.e., at the edges of a 1D topological superconductor (TS), and are seen as spatially localized and well-separated zero-energy quasiparticles. In 1D TS, Majorana modes were first proposed and analyzed by Kitaev in a discrete toy model, the Kitaev chain [1]. The tight-binding Hamiltonian of the Kitaev chain describes spinless electrons and includes nearest-neighbor hopping and the formation of Cooper pairs by *p*-wave pairing, as shown in Figure 1. The Hamiltonian is as follows:

(1) $$H=-\mu \;\sum_{j=1}^{N}c_{j}^{†}c_{j}-\;t\;\sum_{j=1}^{N-1}\left(c_{j}^{†}c_{j+1}+H.c.\right)\;+\;\Delta \;\sum_{j=1}^{N-1}\left(c_{j}^{†}c_{j+1}^{†}+H.c.\right),$$

where *μ* is the electrochemical potential, *t* is the hopping integral, and Δ is the *p*-wave superconductor pairing parameter [16]. The Hamiltonian must be rewritten in the Bogoliubov-de Gennes (BdG) form, which doubles the fermionic degrees of freedom so that each particle state is mirrored by a hole state. Consequently, the BdG Hamiltonian exhibits particle-hole symmetry. Using the Nambu particle-hole basis, the particle and hole creation/annihilation operators are grouped as follows:

(2) $$C^{†}=\left\{c_{1}^{†},\ldots ,c_{N}^{†},c_{1},\ldots ,c_{N}\right\},$$

which leads to the BdG Hamiltonian of the form, as seen below:

(3) $$H=\frac{1}{2}C^{†}H_{BdG}C.$$

For the 1D Kitaev chain, the BdG Hamiltonian reads as follows:

(4) $$H_{BdG}=\left[\begin{array}{cc} H_{0} & \Delta \\ -\Delta^{*} & -H_{0}^{*} \end{array}\right],$$

in which the particle and hole sectors are clearly separated from each other, and in which *H*_0_ contains only the electrochemical potential and hopping terms. The BdG basis can be rearranged into a site-ordered particle-hole basis by using the following:

(5) $$C^{†}=\left\{c_{1}^{†},c_{1},\ldots ,c_{N}^{†},c_{N}\right\},$$

and this arrangement facilitates the implementation of the code for transport simulations [17]. Within this basis, the Hamiltonian of the 1D Kitaev chain becomes the following:

(6) $$H_{KC}=\left[\begin{array}{ccccc} A & B & \; & \; & \;\\ B^{†} & A & B & \; & \;\\ \; & B^{†} & \ddots & \ddots & \;\\ \; & \; & \ddots & A & B\\ \; & \; & \; & B^{†} & A \end{array}\right],$$

which is a matrix of the size 2*N* × 2*N* for an *N*-site Kitaev chain, and where the block-matrices are defined as follows:

(7) $$A=\left[\begin{array}{cc} -\mu & 0\\ 0 & \mu \end{array}\right],\;B=\left[\begin{array}{cc} -t & -\Delta \\ \Delta & t \end{array}\right].$$

Matrices **A** and **B** in (7) can be used directly to find the dispersion of an infinite Kitaev chain [16] by calculating the eigenvalues of the following for each *k* in the first Brillouin zone:

(8) $$H(k)=A\;+\;B\;\exp\left(ik\cdot a\right)\;+\;B^{†}\exp\left(-ik\cdot a\right),$$

where *a* is the site-to-site distance. Additionally, matrices defined in (7) can be employed to construct the Hamiltonian of an *N*-site Kitaev chain and calculate its eigenenergy spectra and wave-functions [17].

The transport properties are calculated using a quantum transport method based on Green’s functions, which is derived from the Keldysh formalism [18,19,20]. The transmission function of finite Kitaev chains is determined using our existing NEGF code for 1D and quasi-1D materials and nanostructures [21,22,23]. Within NEGF, the central quantity is the retarded Green’s function of the device, defined as follows:

(9) $$G^{r}(E)={\left[\left(E+i0^{+}\right)I_{2N}-H_{KC}-\Sigma_{L}^{r}-\Sigma_{R}^{r}\right]}^{-1},$$

where **Σ***_L,R_^r^* matrices are the retarded contact self-energies [24]. The **Σ***_L,R_^r^* matrices account for open boundary conditions between the Kitaev chain and two normal electrodes or leads, denoted as left (*L*) and right (*R*). After calculating the retarded Green’s function, the transmission is found using the following:

(10) $$T(E)=Tr\left[\Gamma_{L}^{r}\;G^{r}(E)\;\Gamma_{R}^{r}\;G^{a}(E)\right],$$

where **G***^a^*(*E*) is the advanced Green’s function of the device, i.e., **G***^a^*(*E*) = [**G***^r^*(*E*)]^†^. The **Γ***^r^_L,R_* = −2 Im**Σ***^r^_L,R_* are the contact broadening matrices for each lead [19], which define tunneling between the device and the contacts and are designated in Figure 1. The contact matrices are considered here by the wide-band limit (WBL) approximation and are energy-independent. The WBL approach amounts to the assumption of metals with a constant density of states (DOS) and a constant metal-chain hopping parameter [24]. The resulting **Σ***^r^_L,R_* matrices contain a single negative purely complex energy parameter, and are suitable for describing contact types ranging from weakly coupled tunneling to normal metal leads [25,26,27,28].

For the described Hamiltonians of the 1D TS, we have to consider the electron and hole degrees of freedom, and the three mechanisms of transport, i.e., direct tunneling, Andreev reflection (AR), and crossed Andreev reflection (CAR) of electrons and holes travelling across the chain [29,30], when calculating the transmission functions. The individual transmission components are as follows:

(11) $$T_{D}^{e,h}(E)=Tr\left[\Gamma_{L}^{e,h}\;G^{r}(E)\;\Gamma_{R}^{e,h}\;G^{a}(E)\right],$$

(12) $$T_{AR}^{e,h}(E)=Tr\left[\Gamma_{L}^{e,h}\;G^{r}(E)\;\Gamma_{L}^{h,e}\;G^{a}(E)\right],$$

(13) $$T_{CAR}^{e,h}(E)=Tr\left[\Gamma_{L}^{e,h}\;G^{r}(E)\;\Gamma_{R}^{h,e}\;G^{a}(E)\right],$$

according to [13,30]. In the site-ordered particle-hole basis defined according to (5) and (6), the $\Gamma_{L,R}^{e,h}$ matrices are of the size 2*N* × 2*N* with non-zero elements only in the upper left and lower right 2 × 2 blocks [17]. The total transmission of the *k*-th mechanism is calculated as the average of the electron and hole transmissions, i.e., $T_{k}=(T_{k}^{e}+T_{k}^{h})/2$ [30]. The overall total transmission depends on the device configuration and will be defined and discussed later, in Section 3.4 and Section 3.5.

While the model defined by (1)–(13) is directly applicable only to finite Kitaev chains, we extend our discussions to realistic TS NWs as well, which is possible due to the qualitative correspondence between the original Kitaev chain model and the Oreg–Lutchyn [9,10] model for TS NWs. Namely, in the Oreg–Lutchyn model a practical realization of a topological superconductor was proposed by combining several ingredients such as time-reversal symmetry breaking by magnetic field, proximity-induced superconductor pairing, and spin–orbit coupling. Despite the differences, both models describe a 1D TS that exhibits Majorana modes at its ends and we expect qualitatively the same behavior in TS NWs as in finite Kitaev chains reported in this paper. This qualitative equivalence is confirmed in the literature by single-subband NEGF simulations of realistic TS NWs [13,31]. In these papers, the NEGF modeling was performed by assuming an effective-mass approximation (EMA) model in which the hopping parameter equals *t* = *ħ*^2^/(2 m^*^a^2^), where *ħ* is the reduced Planck’s constant, m^*^ is the effective mass, and *a* is the spacing on a 1D discretized mesh needed for the numerical implementation of TS NW Hamiltonians. As will be shown later, the TS NW hopping parameter and mesh spacing allow a calculation of NW lengths from the number of sites in the Kitaev chain, and enable an approximate extraction of critical lengths for TS NWs.

## 3. Results and Discussion

The MZMs are characterized by the zero eigenenergy of the two degenerate modes, and by the associated extremely localized WFs at the domain walls. The existence and suitability of these modes for TQC depend on the system parameters (*t*, Δ and *μ*) that can set the conditions in the so-called “sweet spot”. We note that in realistic 1D topological superconductors, i.e., semiconductor nanowires with spin-orbit coupling and proximity-induced superconductivity, one has *t* >> Δ, *μ* [3,10,13], while the case where *t* is comparable to Δ is possible in a Kitaev chain made of coupled quantum dots [6,7]. Therefore, in this work we investigate the effects of different *t*/Δ values, ranging from 1 (“sweet spot”) up to 50 (strong *t*/Δ mismatch in realistic TS NWs). The further we move away from the “sweet spot”, the lower the suitability of the obtained MZMs for TQC becomes. As we will show in the following sections, this problem can be solved by increasing the system length, which reduces MZM hybridization, improves coherence times, and reveals the quantized zero-bias conductance signature. In all these discussions, we define critical or minimum acceptable lengths of finite Kitaev chains, which could be useful for guiding the experiments and designing the system for braiding demonstration [15,16,32].

### 3.1. Parameter Space for MZMs: Majorana Triangle and Majorana Lines

**Figure 2:**
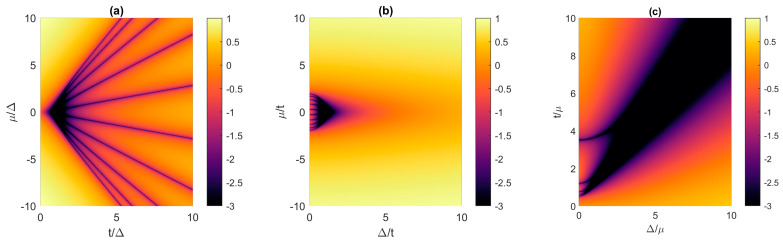
Design spaces considering the spectra of the normalized lowest eigenenergy (*E*_0_/Δ in logarithmic scale) for the 10-site Kitaev chain. The parameter spaces are (**a**) *μ*-*t*, (**b**) *μ*-Δ, and (**c**) *t*-Δ, with the normalization variable being Δ, *t* and *μ*, respectively.

In this subsection, we explore the possible design space of the 1D Kitaev chain by considering the effects of different combinations of *t*, Δ and *μ* on the existence and properties of MZMs. Figure 2 shows the normalized lowest eigenenergies (*E*_0_/Δ) of the 10-site chain in the logarithmic scale as a function of the relationship between *t*, Δ and *μ*. The Δ pairing is fixed in Figure 2a, so that the x- and y-axes show a normalized *t* and *μ*, respectively. In this plot, MZMs with *E*_0_/Δ < 10^−3^ only appear within a triangle formed by the condition |μ/Δ|<2|t/Δ| and by the vertical line drawn at |t/Δ|<1.3. Beyond this “Majorana triangle”, we observe the so-called “Majorana lines” with tightly confined MZMs in the (*μ*, *t*) plane [17,33]. The number of these lines is equal to *N*, and they start at the point (*t*/Δ = 1, *μ*/Δ = 0). For a fixed |t/Δ| outside the Majorana triangle, zero modes are only observed for certain values of the chemical potential. This behavior occurs in chains with an even and an odd number of sites, with the latter exhibiting zero eigenenergies for *μ* = 0 irrespective of the chain length [33]. The oscillatory behavior of *E*_0_/Δ is usually referred to as “Majorana oscillations” [17] and is not desirable in practice as fine tuning of the system parameters is necessary for acceptable MZMs.

A small triangular design space for zero eigenenergies is also reported when Δ and *μ* are varied, with a fixed *t*, as shown in Figure 2b. The occurrence of zero modes is limited by |μ/Δ|<2|t/Δ| and approximately |Δ/t|<2 when *N* = 10. The boundary of the MZM space inclines toward lower chemical potential when Δ is increased, as larger superconductor pairing inclines the Kitaev chain toward a superconducting phase and away from the topological superconducting phase. Consequently, *μ* must decrease to bring the system state closer to the Kitaev point [30,34]. For |Δ/t|<0.8 we again see Majorana oscillations with respect to a change in *μ*, similar to Figure 2a. Finally, when the design space for MZMs is plotted in Δ-*t* space, i.e., when *μ* is held constant and used as a normalization variable, we obtain at a wide zero-mode region extending around the line *t* = Δ and is limited by |t/μ|>0.5. For a very small Δ, we again observe Majorana oscillations, indicating the importance of mitigating their effects in systems with weak superconductor pairing.

**Figure 3:**
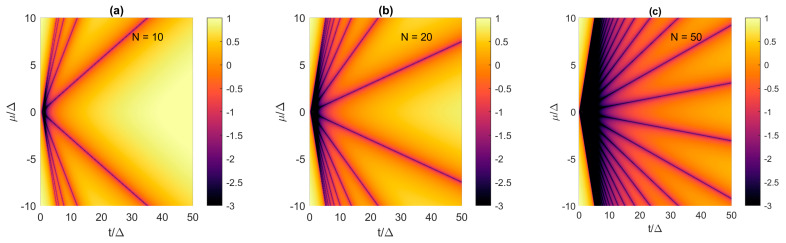
Lowest eigenenergy spectra (*E*_0_/Δ in logarithmic scale) in the *μ*-*t* parameter plane for Kitaev chains with increasing length, i.e., (**a**) *N* = 10, (**b**) *N* = 20, and (**c**) *N* = 50.

The results presented in Figure 2a show that, in the 10-site Kitaev chain, we need *t* < 1.3Δ if we want *E*_0_ < 10^−3^Δ. This requirement seems to be unattainable for realistic TS NWs, since the reported devices exhibit very weak proximity-induced superconductivity with *t* ≈ 40Δ, as determined by Duse et al. using realistic quantum transport modeling of a long Majorana nanowire with an effective-mass tight-binding model [13]. Nevertheless, *t*/Δ mismatch requirement can be relaxed by increasing the chain length. We compare the *μ*-*t* design space for different Kitaev chains having *N* of 10, 20 and 50 in Figure 3a–c, respectively. The hopping and potential are normalized by Δ and the results are shown for a much wider range of hopping amplitudes of up to 50Δ to qualitatively account for realistic TS NWs. Clearly, as the chain length increases, the acceptable parameter range, i.e., “Majorana triangle” with *E*_0_ < 10^−3^Δ, increases as well. This increase occurs continuously in Kitaev chains with an even and an odd number. We can define a critical maximum acceptable value of the hopping parameter (*t_max_*) that divides the design space into feasible and infeasible with respect to MZMs with *E*_0_/Δ < 10^−3^. We obtain *t_max_* ≈ 1Δ for *N* = 10 (Figure 3a), *t_max_* ≈ 3Δ for *N* = 20 (Figure 3b), and *t_max_* ≈ 7Δ for *N* = 50 (Figure 3c). Thus, longer Kitaev chains allow a larger discrepancy between the superconducting pairing and the hopping amplitude, which is advantageous for Majorana NWs. In the following subsections, we define acceptable *E*_0_/Δ values and the associated critical lengths in terms of acceptable coherence times of Majorana modes.

### 3.2. Critical Length of the Kitaev Chain with Parameters Inside Majorana Triangle

In Section 3.1, we have tentatively defined the maximum *E*_0_ = 10^−3^Δ to qualitatively define the design space for MZMs. Here, we define the *E*_0_ limit such that it provides acceptable coherence time (*τ_c_*) of Majorana modes. For plausible TQC, the duration of the Majorana braiding process (*τ_B_*) must be much shorter than the Majorana qubit coherence time that is determined by the splitting or hybridization of Majorana modes [35]. The *τ_c_* is inversely proportional to the tunneling rate between MZMs for a finite Kitaev chain or TS NW [16]. Therefore, we can define *τ_c_* = *ħ*/Γ*_c_*, where Γ*_c_* is the tunneling rate between Majoranas and *ħ* is the reduced Planck’s constant. We approximate the tunneling rate with MZM hybridization in finite wires, taking Γ*_c_* = 2 *E*_0_ so that *τ_c_* → ∞ when *E*_0_ → 0 [35]. If the coherence time is set to an unreasonably large value of *τ_c_* = 1 s, this requires an extremely small *E*_0_ < 3.3∙10^−16^ eV. On the other hand, setting *τ_c_* = 1 μs demands a braiding operation frequency *f_B_* = 1/*τ_B_* >> 1/*τ_c_* = 1 MHz, so that the MZMs can have *E*_0_ < 3.3·10^−10^ eV. For a typical superconductor pairing value of Δ ~ 0.3 meV in proximitized NWs [15,16,36], which is even lower in coupled QD topological systems [7], the coherence time requirement *τ_c_* > 1 μs sets the ground-state energy specification to *E*_0_/Δ < 10^−6^.

**Figure 4:**
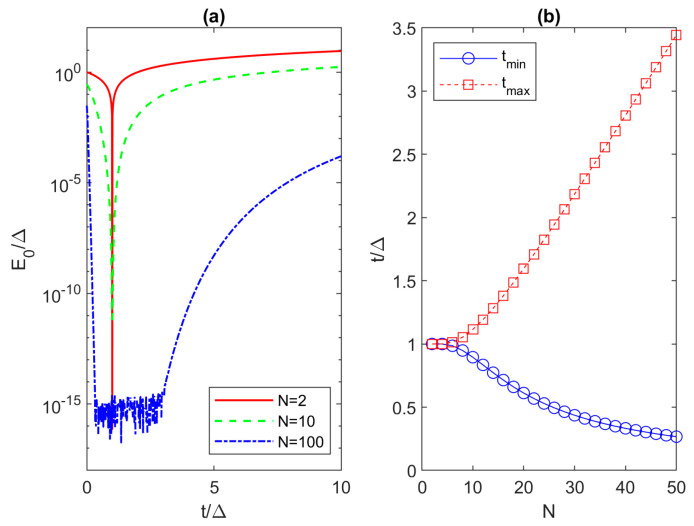
(**a**) Dependence of the minimum eigenenergy on the hopping amplitude for Kitaev chains with *μ* = 0 and *N* of 2, 10 and 100. (**b**) Critical values of the hopping parameter versus Kitaev chain length.

In order to quantify critical hopping, in Figure 4a, we show the dependence of the minimum eigenenergy on the hopping amplitude for different Kitaev chain lengths (*N* of 2, 10 and 100) at *μ* = 0. The logarithmic scale provides a good insight into the *E*_0_ behavior, and we now clearly see that there are two critical *t* values. There is a lower (*t_min_*) and an upper (*t_max_*) limit for acceptable hopping amplitudes if we want *E*_0_ to be a zero-energy mode fulfilling the requirement *E*_0_/Δ < 10^−6^. For the 10-site Kitaev chain, the critical values of *t*/Δ are 0.89 and 1.11 for the lower and upper limit, respectively. Consistent with the expanding design space reported in Figure 3, we also note in Figure 4a that *t_min_* decreases and *t_max_* increases as *N* is increased from 2 to 100, expanding the acceptable hopping values for achieving feasible MZMs with the selected microsecond coherence time. The *t_min_* and *t_max_* are extracted for chain lengths between 2 and 50, and their dependences on *N* are plotted in Figure 4b. While there are hardly any differences between *t_max_* and *t_min_* in chains with the lengths of up to *N* = 6, we report a (nearly) linear dependence of *t_max_* (*t_min_*) on *N* for *N* > 10. By linear fitting in the *N* range between 20 and 50, we obtain the following analytical expressions:

(14) $$t_{\min}/\Delta =-0.011\times N+0.788$$

(15) $$t_{\max}/\Delta =0.062\times N+0.339.$$

These expressions are obtained for the requirement *E*_0_/Δ < 10^−6^, i.e., Majorana coherence time longer than ~1 μs. We point out again that all Kitaev chains with odd *N* have zero modes for *μ* = 0 and their curves would, therefore, look like the minimum-energy part of the *N* = 100 case in Figure 4a, oscillating around the double-precision relative accuracy value available in Matlab 24.2 (R2024a) [37]. Nevertheless, analyzing *t_max_* defined in (15) as the vertical limit of the “Majorana triangle” is applicable to Kitaev chains with an odd number of sites as well.

If we go beyond the *N* range given in Figure 4b, we find *t_max_* = 6.79Δ (13.37Δ) for a Kitaev chain with 100 (200) sites, which means that a larger mismatch between the hopping and the superconducting pairing is possible in longer devices, which in turn significantly reduces the difficulties in experiments. This critical chain length for the parameter space inside the Majorana triangle (*N_c_*) is extracted by linear fitting for *t_max_* with *N* ranging up to 700. We obtain the following expression:

(16) $$N_{c}=\left\lceil 15.190\times \left(t_{\max}/\Delta \right)-3.116\right\rceil ,$$

where ⌈⋅⌉ denotes the ceiling function. Using this analytical expression, we find that *N_c_* = 605 is the shortest Kitaev chain length at which we can have MZMs with *E*_0_ < 10^−6^Δ for the case of large mismatch *t* ≈ 40Δ as in realistic TS NWs. As noted in the Theory and Methodology Section, we expand our discussion to account for TS NWs due to the qualitative equivalence between the original Kitaev chain model and the Oreg–Lutchyn model. Assuming an effective mass of m* = 0.015 *m*_0_ and hopping of *t* = 10 meV, we obtain a lattice spacing of *a* = 15.954 nm according to the EMA NEGF model for proximitized InSb NWs reported in [13]. Therefore, we find the critical NW length (*L_c_* = *a* × *N_c_*) to be ≈9.7 μm. If the proximity-induced superconductivity in Majorana NWs can be improved such that *t*/Δ is reduced from 40 to 10, this would result in a shorter critical length, i.e., *N_c_* = 149 for the 1D Kitaev chain, or *L_c_* ≈ 2.4 μm for a TS NW if we neglect all differences between the models for the Kitaev chain and realistic hybrid nanowires.

Experimentally fabricated proximitized NWs have lengths ranging from 330 nm [36], to 900 nm and 1200 nm, up to 1500 nm [36] and 3000 nm [15], i.e., the fabricated TS NWs with large *t*/Δ mismatch are significantly shorter (<3 μm) than the critical length calculated in the previous paragraph (9.7 μm). For these five experimental device lengths, the corresponding *N*, according to the effective-mass model, are 21, 57, 76, 94 and 188. Using (16), we calculate the following maximum allowable *t*/Δ mismatch values: 1.6, 3.9, 5.1, 6.2, and 12.6. Considering that *t* ~ 40Δ in experimental TS NWs due to the weak proximity effect [3,13,15], it is not surprising that MZMs suitable for TQC have not and cannot be observed in sub-3 μm long nanowires with the current levels of induced superconductivity. Currently, state-of-the-art InSb NWs are ~10 μm long [38], which is comparable to *L_c_* = 9.7 μm. However, the calculated critical length does not come from the simulation of realistic TS NWs which will be conducted in future work, it does not account for disorder, and the initial Majorana braiding frequency is likely to be much slower than 1 MHz. Consequently, the nanowires should be even longer than 10 μm that is currently possible in practice. Therefore, it seems crucial to develop a system where *t* and Δ are matched as close as possible, which would allow the usage of short Kitaev chains. Such system is a chain of quantum dots separated by superconductor islands [6,7]. This solution promises MZMs and would also improve qubit scalability and qubit density in a foreseeable TQC microprocessor based on Majorana modes [39].

Of course, the critical length can be reduced if we relax the zero-energy requirement. If the limit is raised to *E*_0_/Δ < 10^−3^, then *t_min_* would decrease and *t_max_* would increase, as can be inferred from Figure 4a. In turn, the extended range of acceptable hopping amplitudes for MZMs would allow a shorter Kitaev chain for the same mismatch between the hopping and superconductor pairing. For the relaxed limits of *E*_0_, and using a linear fit in the 20–50 chain length range, we obtain new expressions *t_min_*/Δ = − 0.007 × *N* + 0.429 and *t_max_*/Δ = 0.119 × *N* + 0.258, as was calculated for (14) and (15). From these equations we obtain the critical length with the relaxed criterium (*N_cr_*), which is as follows:

(17) $$N_{cr}=\left\lceil 8.433\times \left(t_{\max}/\Delta \right)-2.175\right\rceil .$$

For *t*/Δ of 40 and 10, the resulting critical lengths of the Kitaev chain are 340 and 87 sites, which correspond to the minimum acceptable NW lengths (*L_c_* = *a* × *N_c_*) of 5.4 μm and 1.4 μm, respectively, within the effective-mass tight-binding model for TS NWs from [13]. In comparison to the critical length defined by (16), these critical lengths are ~1.8× shorter, but still far from the experimentally demonstrated devices. We note that the relaxed condition *E*_0_/Δ < 10^−3^ consequently means that the coherence time limit decreases to *τ_c_* ~1 ns, which consequently demands braiding operation frequency *f_B_* = 1/*τ_B_* >> 1/*τ_c_* = 1 GHz. Therefore, the relaxed length requirement is only applicable when the braiding operation speed becomes comparable to the clock frequencies of modern CPUs.

### 3.3. Critical Length of the Kitaev Chain with Parameters Outside Majorana Triangle

**Figure 5:**
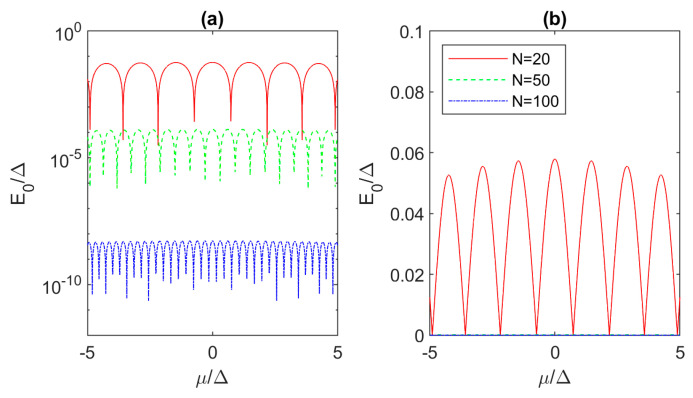
(**a**) Dependence of the minimum eigenenergy on the electrochemical potential for Kitaev chains with *t*/Δ = 5 and *N* of 20, 50 and 100. (**b**) Same as the first panel but in a linear scale.

So far, we have investigated the MZM behavior for *μ* = 0 within the “Majorana triangle” and found critical lengths with respect to the ground-state energy and the Majorana coherence time. Here, we investigate the system with parameters lying outside the Majorana triangle, i.e., we explore the properties for different *μ* for a given mismatch *t*/Δ. First, we set *t*/Δ = 5, which can be understood as e.g., a vertical cut in Figure 3b for *N* = 20. The ground state dependence on *μ*/Δ is plotted in Figure 5a on a logarithmic scale for Kitaev chain lengths of 20, 50 and 100. In all cases we observe an oscillatory behavior of *E*_0_ according to the “Majorana lines” described in Figure 3, which means that suitable MZMs can only be obtained by fine tuning of the electrochemical potential. When *N* increases, *E*_0_ generally decreases, which is beneficial for MZMs and their application in TQC. However, only the 100-site Kitaev chain exhibits all ground energies for which the requirement *E*_0_/Δ < 10^−6^ is satisfied. In the shortest chain (*N* = 20), the ground-state energies reach *E*_0_/Δ ~ 0.058, or *E*_0_ ~ 17 μeV for typical Δ ~ 0.3 meV [15]. For this worst-case scenario and the shortest chain, MZM with the 17 μeV ground-state energy leads to *τ_c_* = 19.4 ps and the required braiding frequency *f_B_* > 52 GHz, which is unreasonable as it demands operation nearly in the terahertz frequency range.

**Figure 6:**
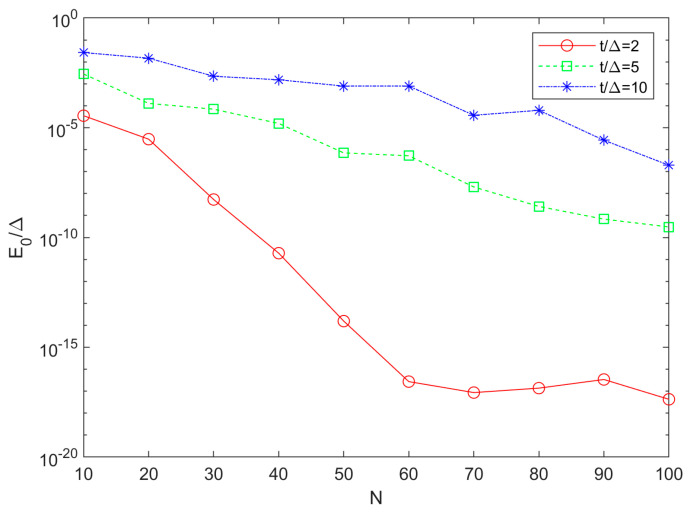
Chain length dependence of the minimum ground-state energy achievable over the entire electrochemical potential range in finite Kitaev chains with *t*/Δ mismatch of 2, 5 and 10. The curves are not perfectly smooth due to numerical limitations in finding energy singularities (see Figure 5a).

Next, we would like to determine the critical lengths for achieving MZMs outside the triangle, assuming the necessary fine-tuning due to oscillations, and considering the given *E*_0_ or *τ_c_* limit. Therefore, in Figure 6 we depict the dependence on *N* of the minimum ground state energy that can be achieved over different *μ* for various *t*/Δ (2, 5 and 10). In general, *E*_0_ decreases with decreasing *t*/Δ towards the “sweet spot” and with increasing chain length, implying that both strategies are plausible to obtain suitable MZMs. For *t*/Δ = 2, *E*_0_ decreases faster with increasing *N* than for hopping values further away from the “sweet spot”. As before, we can set the requirement *E*_0_/Δ < 10^−6^ and find the critical chain length outside the Majorana triangle (*N_co_*) for any *t*/Δ value. The dependence of *N_co_* on *t*/Δ in the range from 2 to 50 is linear and by fitting we obtain the expression

(18) $$N_{co}=\left\lceil 11.524\times (t_{\max}/\Delta )-7.547\right\rceil$$

Thus, if *t*/Δ is equal to 10 and 40, the *N_co_* value is 108 and 454 for the 1D Kitaev chain, respectively. If we apply these findings to realistic hybrid devices, these *N_co_* values correspond to TS NW lengths of 1.7 μm and 7.2 μm. Compared to the critical length of the chain with parameters that lie within the “Majorana triangle”, i.e., for lengths that enable oscillation-free MZMs and microsecond coherence times according to (16), the length requirements outside the triangle as defined by (18) are significantly relaxed. However, the usage of shorter Kitaev chains or TS NWs comes at the expense of the unavoidable fine-tuning of the electrochemical potential in the parameter space outside the “Majorana triangle”.

### 3.4. Conductance in the Kitaev Chain Using NEGF Simulations

The NEGF method enables the investigation of the transmission properties of the Kitaev chain using the Keldysh quantum transport formalism [18,24,40]. The zero-bias conductance is a real-world observable that can be measured in experiments and thus can confirm the existence of MZMs in real topological devices. At *T* = 0 K, the zero-bias conductance is equal to the transmission at zero energy, and the latter can be obtained and analyzed by NEGF calculations [19,41]. In this section, our goal is to determine the critical length of the Kitaev chain for which the zero-bias conductance peak (ZBCP) is observed within 1% of the exactly quantized value of 2*e*^2^/*h*. Again, taking into account the fact that the Kitaev and Oreg–Lutchyn models are qualitatively equivalent, as described in the Theory and Methodology Section, we extend our conclusions on the critical length of the Kitaev chain for ZBCP signatures to TS NWs as well.

A measurement of the local tunneling conductance in an N-S configuration at zero bias should give 2*e*^2^/*h*, and this quantized value comes from the Andreev reflection through the Majorana bound state [4,29]. The AR process starts with an electron entering from the left, which is reflected as a hole, while a Cooper pair (2*e*) is formed in the Kitaev chain and leaving from the right, resulting in a conductance of 2*e*^2^/*h*. In contrast, a non-local zero-bias conductance measurement, i.e., measuring the conductance across the wire in an N-S-N configuration, should give *e*^2^/*h* with symmetric biasing [42]. Our setup focuses on the former, i.e., determining the local conductance on the left-hand side (*G_LL_*). Thus, if MZMs exist, we expect the total zero-bias conductance *G_LL_*(0) at 0 K to be exactly 2*e*^2^/*h*. It is known that AR produces the characteristic quantized ZBCP and is, therefore, crucial for confirming the existence of MZMs in real devices, both in the N-S and N-S-N configurations. Unfortunately, the existence of quantized ZBCPs is not a definite proof of Majorana modes in real devices because nearly quantized conductance peaks can also be induced by trivial states [5,43,44], and the same trivial states can also mimic the closing and reopening of the bulk gap in local and non-local conductances [45].

In our previous work [46], we used NEGF to show how the total transmission, and transmission contributions, inside and outside the topological phase look like. In the topological regime with MZMs, the total transmission at *E* = 0 exhibits a unity peak due to the two MZMs, which are exclusively caused by the Andreev reflection. We have also shown that the transport through the excited states occurs due to direct tunneling and CAR, with the former (latter) being antisymmetric (symmetric) with respect to electron and hole energy sectors. Exactly at the TQPT point, the zero-energy transmission peak disappears. In this case, there are no MZMs, AR and CAR transmission almost completely vanish, and the transport through the “bulk” states is by direct tunneling. Finally, when the system is in the trivial phase, the total transmission exhibits two separate bands due to particle-hole symmetry. The AR and CAR transmissions become weaker, and direct tunneling becomes the dominant transport mechanism in the Kitaev chain. To summarize, AR is dominant in the topological phase, while direct tunneling dominates in the trivial phase. The last mechanism considered, CAR, generates a right-moving Cooper pair within the Kitaev chain by a right-moving electron and left-moving hole at the second interface, i.e., the right-hand end of the Kitaev chain. The CAR process is weak, irrespective of the chain configuration. Based on these features of transmission across the Kitaev chain, we can determine under which conditions the system exhibits quantized ZBCP due to topological MZMs.

### 3.5. Critical Length for ZBCP Signature

In the previous subsections, we determined the length requirements in terms of coherence time via *E*_0_. Here, however, we calculate and discuss the minimum acceptable lengths of the Kitaev chain that makes visible the characteristic MZM conductance signature, i.e., *G_LL_*(0) = 2*e*^2^/*h*. According to the derivation in [31], the total local conductance *G_LL_* is proportional to the following total transmission, as seen below:

(19) $$T_{TOT}(E)=T_{D}(E)+T_{CAR}(E)+2\times T_{AR}(E),$$

where the individual contributions are defined in Section 2. For the contact broadening, we assume in all simulations $\Gamma_{L}=\Gamma_{R}=0.1\Delta ,$ and this value describes a weakly coupled tunneling contact. At 0 K, the zero-bias conductance is proportional to the zero-energy transmission, i.e., *G_LL_*(0) = *e*^2^/*h* × *T_TOT_*(0), so that the Majorana signature in *T_TOT_*(*E*) is the zero-energy transmission of two.

**Figure 7:**
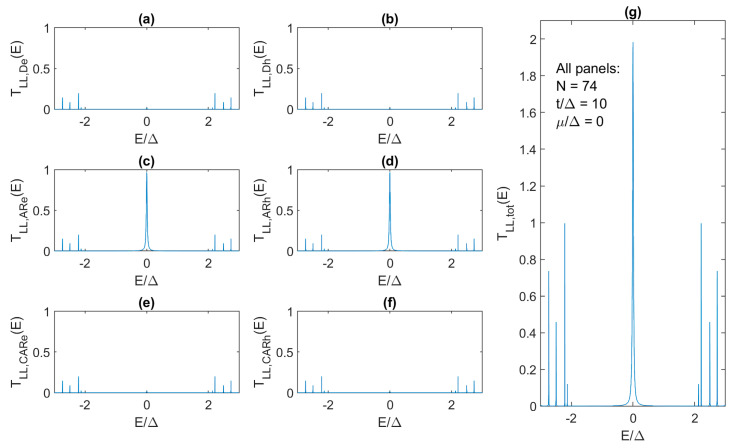
Transmission characteristics for direct tunneling of (**a**) electrons and (**b**) holes, Andreev reflection of (**c**) electrons and (**d**) holes, crossed Andreev reflection for (**e**) electrons and (**f**) holes, with (**g**) showing the total transmission as defined in the text. *N* = 74, *t*/Δ = 10, *μ*/Δ = 0.

The critical Kitaev chain length, in terms of the quantized ZBCP signature, is first discussed for the case where *t*/Δ = 10 and *μ*/Δ = 0. We have previously obtained *N_c_* = 149 and *N_co_* = 108 and, indeed, for *N* = 150 we obtain the expected quantized *G_LL_*(0) = 2*e*^2^/*h*. However, we also obtain an almost exactly quantized ZBCP even for much shorter chains. As shown in Figure 7, the ZBCP height is equal to ≈1.98 in the 74-site Kitaev chain. For *N* = 74 and *t*/Δ = 10, Andreev reflection is the dominant process that produces *T_TOT_*(0) ≈ 1.98 for the ZBCP despite the significant *t*/Δ mismatch (Figure 7c,d), while CAR (Figure 7e,f) and direct tunneling (Figure 7a,b) are negligible in the topological phase (ensured by *μ* = 0 < 2*t*). Therefore, we can achieve a nearly perfectly quantized conductance signature within 1% of the exact value in a chain whose length is ~2× smaller than *N_c_* defined in (16) and ~33% smaller than *N_co_* defined in (18). This finding means that the condition for an almost perfectly quantized ZBCP signature is much more relaxed than the length requirement for a coherence time of ~1 μs.

**Figure 8:**
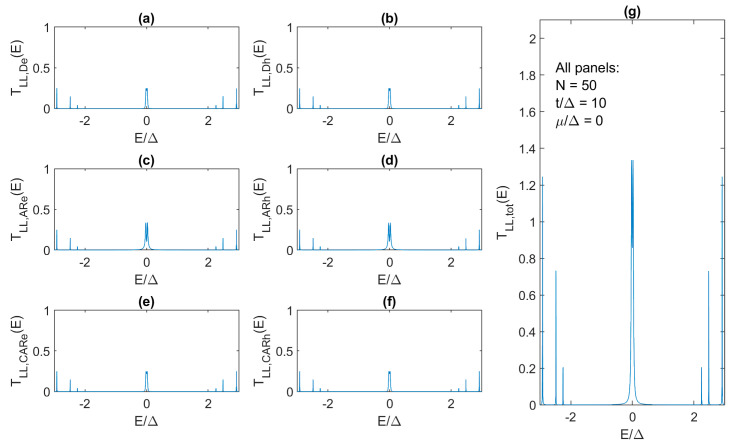
Same as in Figure 7 but for the 50-site Kitaev chain with *t*/Δ = 10 and *μ*/Δ = 0.

By increasing the chain length to more than 74 sites, *T_TOT_*(0) moves closer to 2. For example, we obtain *T_TOT_*(0) that equals 1.998 for the 84-site Kitaev chain, i.e., the ZBCP is within 0.1% of the quantized value. In contrast, *T_TOT_*(0) decreases in shorter chains and a single MZM hybridizes into two separate levels with two separate transmission peaks, as shown in Figure 8, for *N* = 50. In the 50-site chain with *t*/Δ = 10 and *μ*/Δ = 0, the maximum total transmission is only 1.34 due to the much weaker (max. *T_AR_*(*E*) ~ 0.34) and hybridized Andreev reflection (Figure 8c,d). When the length is reduced to *N* = 50, both direct tunneling (Figure 8a,b) and CAR (Figure 8e,f) become stronger, leading to the maximum *T_TOT_*(*E*) ~ 0.25 at and around zero energy. After going through a range of different chain lengths from *N* = 84 downward, we find *N* = 58 to be the shortest chain length at which Majorana hybridization and conductance peak separation do not occur. Nevertheless, in this case, *G_LL_*(0) is not perfectly quantized and is equal to only 1.61 × *e*^2^/*h*.

**Figure 9:**
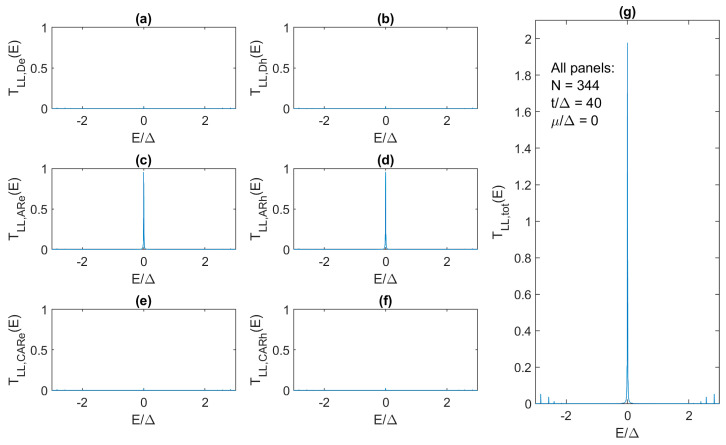
Transmission characteristics as in Figure 7 but for the 344-site Kitaev chain with *t*/Δ = 40 (large mismatch as in realistic TS NWs) and *μ*/Δ = 0.

Qualitatively identical behavior is also reported for the case of a much larger mismatch between the hopping and superconductor pairing. For *t*/Δ = 40, we find *N* = 344 as the Kitaev chain length for which we are within 1% of the quantized ZBCP, i.e., *G_LL_*(0) = 1.98 *e*^2^/*h* (Figure 9g). This chain length corresponds to a 5.5 μm long proximitized nanowire [13], which means that perfect ZBCPs cannot be observed even in defect-free NWs if they are less than 3 μm long, as reported experimentally thus far [3,15,36]. In the 344-site Kitaev chain, direct tunneling and CAR are effectively turned off (Figure 9a,b,e,f), and only AR (Figure 9c,d) contributes to total transmission and ZBCP. Going in the other direction, i.e., away from a large mismatch and towards the “sweet spot” when *t*/Δ = 1, we obtain *G_LL_*(0) ≈ 2*e*^2^/*h* even for the shortest chain with *N* = 2 indicating the great promise of the coupled quantum dot approach for short topological chains [6,7,8].

When we use the obtained critical lengths for several different *t*/Δ mismatch values larger than 10, we observe a nearly linear trend describing the behavior of the critical length for the quantized conductance signature (*N_cq_*), for which the equation is as follows:

(20) $$N_{cq}=\left\lceil 8.827\times (t/\Delta )-10.065\right\rceil ,$$

assuming *G_LL_*(0) within 1% of 2*e*^2^/*h*. This expression stresses the fact that the length requirement for quantized ZBCPs is easier to fulfill than the requirement for coherence time. In other words, we must be cautious because we can measure a clear ZBCP Majorana signature (within 1%) while the coherence time is orders of magnitude shorter than ~1 μs, demanding unrealistically high and impractical braiding speeds.

**Figure 10:**
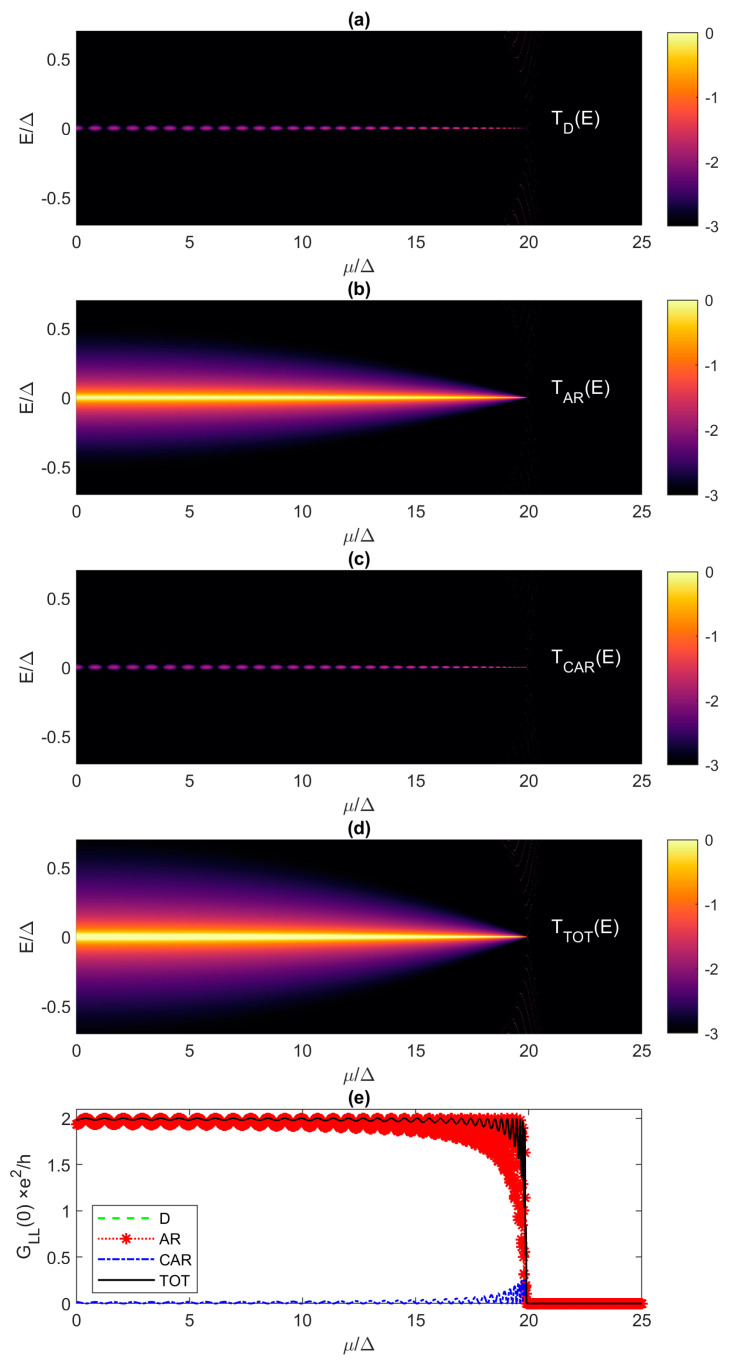
Transmission spectra versus energy and electrochemical potential for (**a**) direct tunneling, (**b**) Andreev reflection, (**c**) crossed Andreev reflection, and (**d**) total transmission. The transmission spectra are plotted in the logarithmic scale. (**e**) Total local zero-bias conductance and main contributions for varying *μ*. *N* = 74, *t*/Δ = 10.

So far, we dealt with transmission inside and outside the “Majorana triangle” from Figure 3, but assuming a single value of the electrochemical potential, i.e., *μ* = 0. Here, we evaluate the transmission and conductance spectra for different electrochemical potentials, which allows us to study Majorana oscillations outside the “Majorana triangle”. As already shown, we are within 1% of the exactly quantized ZBCP for *t*/Δ = 10 if the Kitaev chain is at least 74-site long. For this case, Figure 10 reports the transmission spectra as a function of energy and *μ* for *T_D_*, *T_AR_*, *T_CAR_* and *T_TOT_* in Figure 10a–d, respectively, while Figure 10e shows the total *G_LL_*(0) as a function of *μ* compared against individual contributions to the total conductance. Direct tunneling and CAR exhibit a clear oscillatory behavior, as *μ* increases from 0 to 20Δ, while AR appears to be equally strong regardless of potential, with a much stronger broadening than for the *T_D_* and *T_CAR_* transmission functions. For *μ* > 20Δ, beyond the TQPT point, the system is in the trivial phase where there are no transmission signatures in the selected energy range. Figure 10e shows that *G_LL_*(0) oscillates with increasing *μ*, because the Andreev reflection exhibits oscillations and reaches exactly two periodically, depending on *μ*. Therefore, an exactly quantized ZBCP is possible even for *t*/Δ = 10 and *N* = 74, but only if the electrochemical potential can be very finely tuned within ~0.1Δ, which is difficult to achieve in practice.

**Figure 11:**
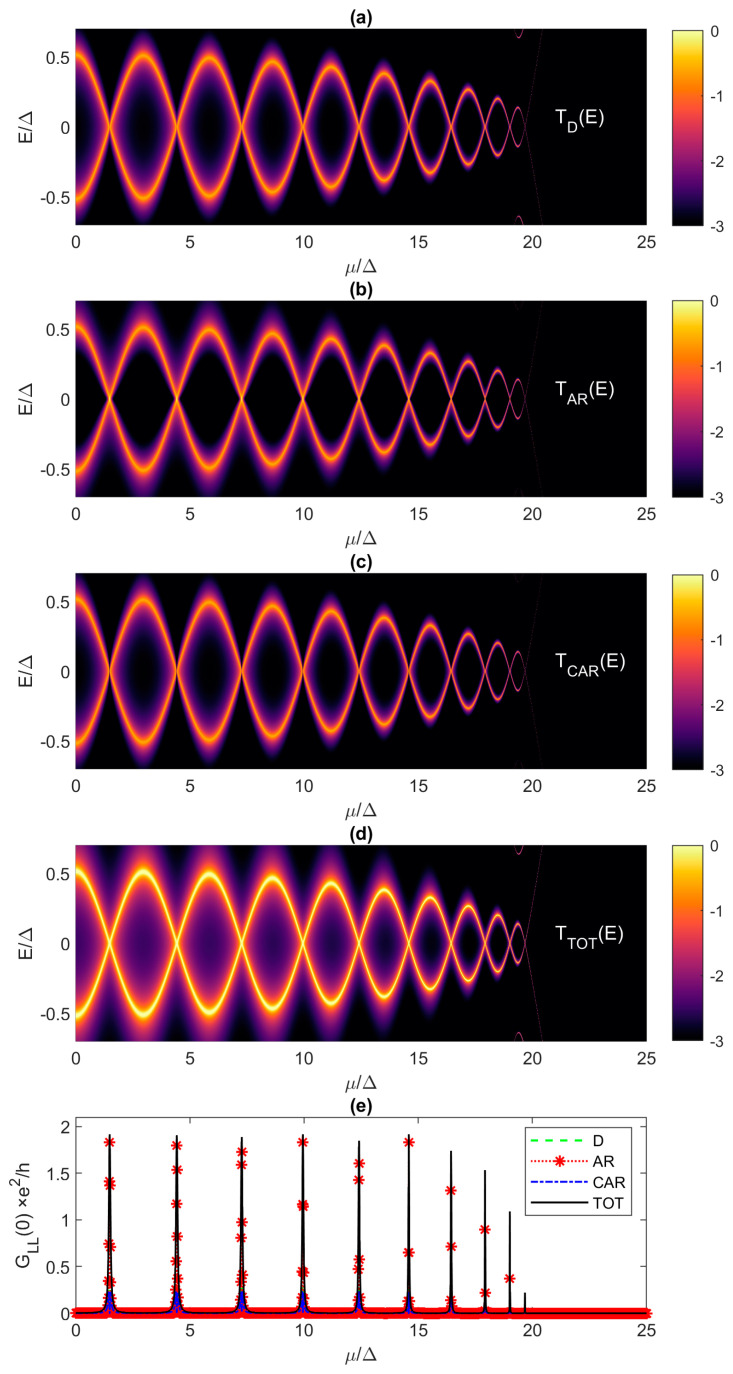
Transmission spectra and local conductance as in Figure 10 but for a shorter Kitaev chain with *N* = 20 and the same mismatch *t*/Δ = 10. The transmission spectra are plotted in the logarithmic scale.

We have already observed that decreasing the chain length leads to a decrease in ZBCP and MZM hybridization (see Figure 8 for the case of *N* = 50). Next, we illustrate in Figure 11 the evolution of the transport properties when *N* is reduced down to only 20 sites, leaving the mismatch unchanged at *t*/Δ = 10. In the 20-site Kitaev chain, all three transmission mechanisms exhibit oscillatory behavior, with crossing points occurring at *E* = 0 for very specific *μ* values. The MZMs are present exactly at these zero-energy crossings and the oscillations are due to the non-zero overlap between the two Majorana wave-functions [17]. In contrast to the longer chain (*N* = 74), all transport mechanisms contribute equally to the total transmission in the 20-site chain, except exactly at the crossing point, as shown in the conductance plot. The zero-bias conductance (Figure 11e) oscillates and exhibits very sharp peaks when *μ* increases from 0 to 20Δ, which is due to peaks in *T_AR_*(0). Exactly at the parity crossing points, contributions of direct tunneling and CAR are negligible. Therefore, nearly quantized ZBCPs are possible in the much shorter 20-site Kitaev chain, but the sharp peaks indicate that extremely precise tuning of the electrochemical potential would be required to obtain *G_LL_*(0)~2*e*^2^/*h*, which seems implausible from an experimental point of view for a practical realization of a TQC processor.

For the data presented in Figure 10 and Figure 11, we note the similarity between the NEGF simulation results for these finite Kitaev chains and the EMA NEGF analysis of TS NWs of different lengths reported in [13,31]. The similarity includes Majorana oscillations in short devices and quantized ZBCPs in longer nanowires, demonstrating the equivalence of the Kitaev and Oreg–Lutchyn models in the transport physics, and the existence of critical lengths in TS NWs similar to those we reported for finite Kitaev chains. Although we expect rather similar results, finding the exact critical lengths for realistic hybrid devices using NEGF simulations will be the topic of future work.

## 4. Conclusions

In this work, we have analyzed the ground-state energy spectra and transport properties of finite Kitaev chains in order to define possible design spaces for Majorana zero modes that are suitable for TQC. The effect of varying *t*, *μ* and Δ on the “Majorana triangle” is investigated for Kitaev chain lengths ranging from a few sites up to several hundred sites. Due to the correspondence of the Kitaev and Oreg–Lutchyn models, our conclusions are also generally applicable to TS NWs. We found that we can define critical or minimum acceptable chain lengths (*N_c_*, *L_c_*) that lead to oscillation-free MZMs with coherence times in the microsecond range, and that this critical length depends almost linearly on the *t*/Δ mismatch. In realistic TS NWs with *t*/Δ ~ 40, we obtain *N_c_* = 605 or *L_c_* ≈ 9.7 μm, implying that the experimental TS NWs reported so far cannot host plausible MZMs with reasonable coherence time and without oscillations. If the mismatch can be reduced to about *t*/Δ ~ 10, we obtain *N_c_* = 149 or *L_c_* ≈ 2.4 μm, which brings the critical Kitaev chain length close to fabricated devices with reported lengths of 330 nm to 3 μm. On the other hand, NEGF simulations provide insights into transmission mechanisms that include direct tunneling and Andreev processes, and allow the calculation and analysis of ZBCP at 0 K. Using these results, we determine critical Kitaev chain lengths for the observation of ZBCPs that are almost perfectly quantized (within 1%) at 2*e*^2^/*h*. For *t*/Δ = 40, we find *N_c_* = 344 or *L_c_* ≈ 5.5 μm, indicating that perfect ZBCPs cannot be observed even in defect-free NWs if they are less than 5.5 μm long. If the mismatch can be decreased to *t*/Δ ~ 10, we obtain a much shorter critical length of *N_c_* = 74 or *L_c_* ≈ 1.2 μm. The length requirement with respect to ZBCPs is less stringent than the requirement resulting from *E*_0_ and *τ_c_* limits. Therefore, we can observe a nearly perfect ZBCP as a Majorana signature in experiments, even though coherence time is not sufficiently long. While our work neglects the effects of finite temperature and disorder, our results, unfortunately, indicate additional difficulties for the TS NW route for MZMs. Nevertheless, the prospects for Majorana qubits and fault-tolerant TQC are still promising, thanks to the quantum-dot approach that is more resilient to disorder- and length-scaling effects.

## Data Availability

The original contributions presented in this study are included in the article. Further inquiries can be directed to the corresponding author.
